# The Discovery of Endoplasmic Reticulum Storage Disease. The Connection between an H&E Slide and the Brain

**DOI:** 10.3390/ijms22062899

**Published:** 2021-03-12

**Authors:** Francesco Callea, Valeer Desmet

**Affiliations:** 1 Bugando Medical Centre, Department of Molecular Histopathology, Catholic University Health Allied Sciences, P.O. Box 1464 Mwanza, Tanzania; 2 Department of Pathology, Catholic University of Leuven, B-3000 Leuven, Belgium; v.desmet@telenet.be

**Keywords:** endoplasmic reticulum storage disease, alpha-1-antitrypsin deficiency, hereditary hypofibrinogenemia hepatic storage

## Abstract

The revolutionary evolution in science and technology over the last few decades has made it possible to face more adequately three main challenges of modern medicine: changes in old diseases, the appearance of new diseases, and diseases that are unknown (mostly genetic), despite research efforts. In this paper we review the road travelled by pathologists in search of a method based upon the use of routine instruments and techniques which once were available for research only. The application to tissue studies of techniques from immunology, molecular biology, and genetics has allowed dynamic interpretations of biological phenomena with special regard to gene regulation and expression. That implies stepwise investigations, including light microscopy, immunohistochemistry, in situ hybridization, electron microscopy, molecular histopathology, protein crystallography, and gene sequencing, in order to progress from suggestive features detectable in routinely stained preparations to more characteristic, specific, and finally, pathognomonic features. Hematoxylin and Eosin (H&E)-stained preparations and appropriate immunohistochemical stains have enabled the recognition of phenotypic changes which may reflect genotypic alterations. That has been the case with hepatocytic inclusions detected in H&E-stained preparations, which appeared to correspond to secretory proteins that, due to genetic mutations, were retained within the rough endoplasmic reticulum (RER) and were deficient in plasma. The identification of this phenomenon affecting the molecules alpha-1-antitrypsin and fibrinogen has led to the discovery of a new field of cell organelle pathology, endoplasmic reticulum storage disease(s) (ERSD). Over fifty years, pathologists have wandered through a dark forest of complicated molecules with strange conformations, and by detailed observations in simple histopathological sections, accompanied by a growing background of molecular techniques and revelations, have been able to recognize and identify arrays of grotesque polypeptide arrangements.

## 1. Introduction

The term endoplasmic reticulum storage disease (ERSD) was introduced into the medical literature after discovering that, similarly to alpha-1-antitrypsin (AAT) [1], due to a genetic mutation, another acute phase protein, fibrinogen, could undergo misfolding within the endoplasmic reticulum (ER) instead of being exported into the blood [2]. The two related diseases were designated alpha-anti-trypsin-deficiency (AATD) and hereditary hypofibrinogenemia with hepatic storage (HHHS). Together with other intracellularly or extracellularly misfolded proteins, ERSD are classified as conformational diseases [3] (Table 1), and are included together with mitochondrial, lysosomal, and peroxisomal diseases into the group of pathologies of cell organelles [4], and into the metabolic disorders and pathologies of the liver [5].

The uniqueness of these two diseases resides in the fact that both were described by pathologists from the evaluation of H&E-stained slides, yet they were easily overlooked on clinical grounds. Indeed, AATD individuals in the heterozygous state may have normal AAT serum levels [6], and HHHS patients, despite the low plasma fibrinogen levels, may not show overt signs of coagulopathy [2,7]. Both ERSD cause chronic liver disease and cirrhosis. The hallmark of both diseases is the presence of cytoplasmic inclusions in liver cells visible with a light microscope on H&E-stained slides. An ad hoc protocol made up by stepwise investigations including special stains, immunohistochemistry (IHC), electron microscopy (EM), molecular histopathology, gene sequencing, and 3D modelling has fully explained the pathomorphogenesis of the storage process and has led to the recognition of these new disease entities.

In this paper we review the diagnostic methodology; and we provide an explanation as to how and why H&E-stained slides can provide the correct diagnosis, and by applying the ad hoc protocol, have turned out to be equivalent to molecular genetic analysis when the latter is not available [6,7,8].

### 1.1. The Amazing Universe of the Cell Microstructure and Function

In 1665, Robert Hooke, a botanist, wrote: “By examining with my microscope, sections of cork, I have been able to discover rather easily that the cork is composed of a mosaic of ‘empty polygonal spaces,’ whilst I haven’t reached any certainty with animal tissue. However, it is not unlikely that an observer, some day, with the help of a better microscope could discover them in thinner histological sections” [9]. In 1838, Matthias Jacob Schleiden, the lawyer–botanist–physician, coined the term cell for those microscopic units that he had found in all plants [4]. In the same era, Theodor Schwann, professor of anatomy and physiology at the University of Leuven, discovered the same microscopic units in animal tissues. During a friendly discussion one evening, the two scientists concluded that finally biology had a sole elementary unit, the cell, and forwarded the cellular theory [4].

Nineteen years later, Rudolph Virchow, professor of pathology in Berlin, prophetically baptized the cell as “the elementary patient” endowed with respiratory (mitochondria), digestive (lysosomes), and locomotor (contractile filaments and microtubules) apparatuses, and excretory and sensory (skin and cell membrane) systems. Based upon physiological and pathological histology, Virchow figured out pathology as “physiology with obstacles,” and invented the cellular pathology [10].

### 1.2. Endoplasmic Reticulum as a New World in the Cell Universe

At the end of the 19th century, an Italian pathologist, Camillus Golgi, with rudimentary techniques and a self-made stain, discovered within some neurons’ cytoplasm the “internal reticular apparatus,” a crucial component of the labyrinthine network of channels that are the theater of the intracellular protein traffic—the endoplasmic reticulum (ER) [11]. The cell organelle was called Golgi complex or Golgi apparatus and granted the discoverer with the Nobel prize in 1906.

The Golgi apparatus turned out to be for light traffic, for sorting out and addressing proteins, as demonstrated over the 20th century by histo-autoradiography [12], electron microscopy (EM) [13], fractioned ultracentrifugation [14], electron spray mass ionization [15], and imaging mass spectrometry [16].

The pioneering work on the anatomical distribution of the rough-ER (RER) in hepatocytes by Ma and Biempica [17]; on protein synthesis by Farquhar and Palade [18], Sabatini [19], and Gilmore [20,21]; on the defective translocation of the most frequent AAT mutant (proteinase inhibitor (Pi) Z) from the RER to the smooth ER (SER) in AATD [22]; and more recently, on endoplasmic reticulum associated degradation (ERAD) [23] and its quality control regulation [24], have largely contributed to the understanding of the genetic and molecular mechanisms of the defective export of secretory proteins and have led to the concept of endoplasmic reticulum storage disease (ERSD) [2,6].

### 1.3. The Biosynthesis of Glycoproteins in General

Glycosylation of proteins is a stepwise process by which monosaccharides are covalently bound to a polypeptide chain during its elongation, intracellular transport, and excretion, resulting in a product termed glycoprotein [25]. This mechanism requires a protein acceptor, a sugar donor, and enzymes (glycosyltransferases) which catalyze the transfer. Genetic regulation of the process occurs at the level of the protein acceptor and glycosyltransferases. The sugar donor is not under direct genetic control; however, the enzyme-substrate specificity is very high [26].

The attachment of the first sugar (“core” glycosylation) takes place in a special way: a soluble lipid, the dolichol phosphate, plays the role of intermediate between the sugar nucleotide and the peptide. That first accepts the sugar, and subsequently, when the side chain is completed, transfers the “core” oligosaccharide en bloc to the protein [27]. Glycosylation steps are common to all glycoproteins, whether secretory, membrane, or cellular. The amount of carbohydrate content varies in mature proteins and depends upon the glycosylation steps along the secretory pathway and the trimming and elongation processes of glycopeptides [28]. The content of oligosaccharides in AAT is much higher than in fibrinogen.

The biosynthesis of glycoproteins starts at the level of polyribosomes attached on ER membranes (RER). During the elongation of the peptide chain (translation), while the latter is still in the membrane, the “core” glycosylation takes place (core glycosylation is a co-translational event). At this early stage, a sorting out mechanism (co-translational insertion signal, co-translational cleavage, halt transfer signal) determines the fate of the nascent glycopeptide: discharge and segregation into the lumen of the RER or insertion into membranes [19]. During the vectorial discharge, the polypeptide chain gets into contact with enzymes associated with membranes or present within the cisternal space [12], responsible for proximal glycosylation, disulphide bridge formation, hydroxylation, and partial proteolysis. The resulting modifications influence the tertiary structure of the secretory proteins, which, once assumed, renders the proteins impermeant and their segregation irreversible [29]. However, misfolded proteins can be unfolded and retrotranslocated into the cytosol for degradation via autophagy or proteasome pathway [23,24,28,29,30]. Next, the intracisternal proteins and those inserted into membranes are transferred through the rough and smooth ER (SER), where further glycosylation is performed by membrane-associated glycosyltransferase. Subsequently, they reach the Golgi complex, where a new crucial sorting-out mechanism differentiates proteins destined to extracellular export from those destined to intracellular organelles. That is due to the recognition by the proteins of specific endomembrane receptors located in the Golgi endoplasmic reticulum lysosome (GERL) area, where lysosomes originate [12,19].

In the Golgi complex, glycoproteins acquire the completion of the carbohydrate chain by addition of the terminal sugar, *N*-acetylneuraminic acid (sialic acid). Finally, they are driven, within secretory vesicles with the cooperation of the microtubule system, towards the cell surface where fusion of the vesicle membrane with the cell membrane occurs. At this time, the internalized proteins are discharged into the blood or to the extracellular space, whilst the proteins to be incorporated into membranes remain there and contribute to the reconstitution of the integrity of the plasma membrane [19].

The above scheme works for cells specialized in protein production for export, such as hepatocytes. Some variations are known to occur in other cells [31,32].

## 2. Apha-1-Antitrypsin Deficiency (AATD): The Prototype of ERSD

Alpha-1-antitrypsin (AAT), the major component of alpha-1-globulins, is the major serum proteinase inhibitor (Pi), especially active against leucocyte elastases. AAT is also an acute phase reactant, capable of raising its serum level up to quadruple the normal level under conditions of clinical stimulation [33].

The discovery of AAT deficiency (AATD) represents one of the most fascinating examples of scientific intuition, logical clinical reasoning, and research methodology. During an influenza epidemic in 1963 in Sweden, a number of hospitalized patients with pneumonia showed a peculiar abnormality in the serum electrophoresis consisting of a flat or deeply depressed alha-1 globulin pick [34]. Initially, this profile was considered as an effect of the viral infection. However, a young pneumologist, Sten Eriksson, did notice that several patients were carrying the same surname. That suggested to him to check the clinical records from the archives. To his astonishment, he found that the same electrophoretic profile recurred in the absence of epidemics in members of the same families, which was sufficient for a bright mind to conclude that AAT deficiency was a hereditary, congenital, and familial disease [34,35]. Upon isoelectric focusing (IF), AAT from those patients showed the slowest electrophoretic mobility and for that reason was called Z by the last letter of the alphabet. For several years, IF has been a useful diagnostic test to distinguish homozygous from heterozygous individuals and to identify more than hundred polymorphic variants [36].

**Figure 1:**
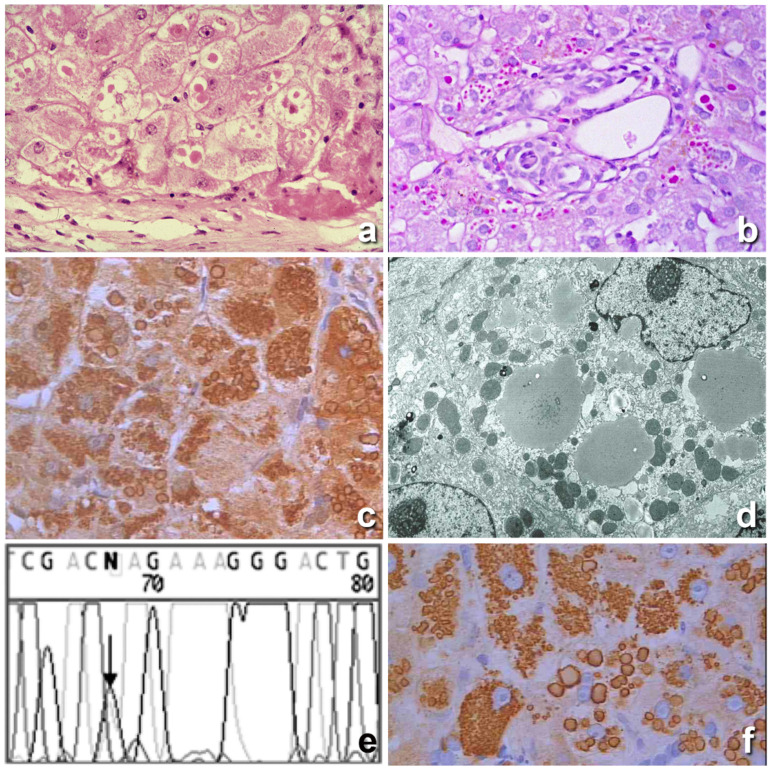
Histological section from a Pi ZZ liver. Hepatocytes contain cytoplasmic, round eosinophilic inclusions in a periportal area (**a**), H&E × 40). The eosinophilic cytoplasmic inclusions are strongly positive on PAS staining after amylase digestion. (**b**), PAS-diastase (PASD) × 40). The inclusions were positively stained with a polyclonal alpha 1 anti-trypsin (AAT) antibody (**c**), immunostaining × 40). The electron microscopy (EM) picture shows amorphous fluffy semielectron dense material corresponding to AAT within dilated cisternae of the rough endoplasmic reticulum (RER). No material is observed within the smooth endoplasmic reticulum (SER) or Golgi apparatus (**d**), EM × 16,000). The chromatogram profile shows the sequence of SERPINA 1 Exon V flanking the p.Glu342Lys (ZAAT mutation) (**e**). The AAT inclusions were positively stained with the monoclonal ATZ11 antibody, specific for the Z variant of AAT (**f**), immunostaining × 40).

Six years later, Harvey Sharp, a pediatrician in Minneapolis, did observe the same electrophoretic pattern in children affected by cryptogenic liver cirrhosis and low serum AAT levels [17]. Sharp performed a liver biopsy from the diseased children and found within hepatocytes peculiar inclusions appearing as round eosinophilic globules (Figure 1a) that were strongly positive in Periodic Acid Scif -diastase (PASD) stain (Figure 1b). The PAS after diastase digestion (PASD) works as a staining for glycoprotein. The strong positivity of AAT globules was due to its high content of carbohydrates. In view of the low serum AAT levels in those patients, Sharp correlated the two observations and immunized rabbits by using as antigen the purified human AAT and obtained a polyclonal rabbit anti-human AAT antibody. As expected, the antibody reacted positively with the liver tissue inclusions (Figure 1c) thus proving their identity. In the same study the electron microscopic (EM) examination revealed the presence of the stored protein within dilated cisternae of the ER (Figure 1d) [1].

In 1976 it was shown that the Z AAT was carrying a molecular abnormality consisting of a Glu–Lys substitution [37], and in 1982 Carrell et al. published the structure and variation of human AAT, demonstrating that the nucleotide replacement was located at position 342 of exon V of the AAT gene (Figure 1e) [38].

In 1984 an EM study on a large series of both homozygous (Pi ZZ) and heterozygous (Pi MZ) AATD individuals [22], could definitely demonstrate that the storage phenomenon was occurring in the very early stage of synthesis, and that the aggregation of the mutant protein was taking place exclusively in the RER (Figure 1d). No AAT was detected in the SER or Golgi apparatus, thereby indicating a defective translocation from the RER along the secretory pathway. The crystal structure (3D modelling) of the Z protein could explain the mechanism of aggregation as due to a unique molecular interaction between the reactive center loop of one molecule and the gap in the A-sheet of another [8,39].

In 1975, Jeppsson et al. [40] were able to extract and purify the ZAAT from a homozygous cirrhotic liver and to raise the monoclonal ATZ11 antibody, capable of specifically interacting with Pi Z AAT [41] and detecting ZAAT molecules in the polymerized form [42]. The antibody was used first in an Elisa system to screen the Swedish population [41], and later on in an immunohistochemical (IHC) test that definitely proved that PASD positive hepatocytic inclusions corresponded indeed to the Z variant of AAT (Figure 1f). That was the first time that the genetic diagnosis of AATD was made on tissue sections [43].

The above methodology has allowed to distinguish the storing from the non-storing deficiency variants of AAT. The latter indeed are not predisposing to the development of liver disease [44] thus indirectly suggesting that the storage process is toxic per se [8,45].

**Figure 2:**
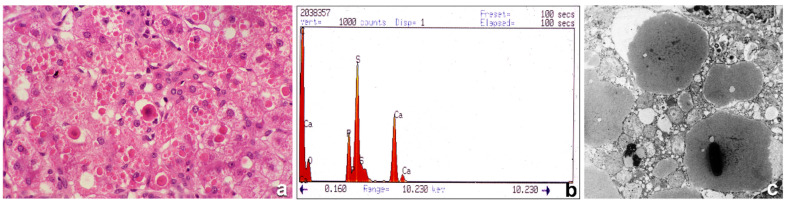
Histological section from a PiMmalton cirrhotic liver. Hepatocytes contain large numbers of cytoplasmic eosinophilic inclusions. A few inclusions are centered by a dark basophilic material (**a**), H&E × 60). The electron microphotograph shows electrondense crystal-like or granular precipitates inside the AAT intracisternal inclusions of three adjacent hepatocytes (**c**), EM × 20,000). X-ray microanalysis for metal detection shows two picks corresponding to calcium. The electron beams were centered on electron dense precipitates under the EM (**b**), X-ray).

Even more interesting was the detection of dark basophilic material inside the eosinophilic AAT globules (Figure 2a). On EM, AAT inclusions contain electrondense precipitates in the form of crystalline structures or granular material (Figure 2c). The intraglobular dark precipitates turned out to correspond to calcium precipitates, as demonstrated by von Kossa staining for calcium [8], and by electron probe X-ray microanalysis method according to Fernandez-Segura et al. [46] (Figure 2b). Calcification of AAT inclusion bodies does not occur with Z and Siiyama, which have become the hallmark of the Mmalton AAT variant, whose genetic abnormalities reside at exon II where a critical 52Phe deletion is located [47].

There is an additional variant of AAT that is stored within hepatocytes and that equally predisposes to liver cirrhosis, the Siiyama, whose mutation, Ser53Phe, has been localized at exon III [48]. By using the above histopathological methodology, the three variants can be distinguished under the light microscope: all of them present the same H&E appearance and the same PASD positivity and immunoreactivity with polyclonal AAT antibodies, but Mmalton and Siiyama do not react with the monoclonal anti-Z-AAT antibody and only Mmalton AAT inclusions present calcium precipitates [8]. 3D protein analysis has allowed us to predict more severe misfolding of the Mmalton molecule as compared to Z and Siiyama, which could trigger anomalous interaction with endoplasmic reticulum chaperons, namely, calcium binding proteins [8].

As a sort of scientific butterflies seduced by flowers along our way, we have wandered through a dark magic forest of complicated serine molecules and made detailed descriptions of their strange molecular malformations. Studies on the growing background of multiplying molecular techniques and revelations, the observations have allowed to recognize and identify the array of grotesque polypeptide acrobatics in simple histopathological stains. Most of these observations were challenges we met in diagnostic and research work, with the recurring question: “what could be the reasons for this lesion looking like this?” [49].

The following paragraph will be dealing with a newly discovered disease similar to AATD, regarding a protein of a major hematological interest, the fibrinogen, that was so unexpected and apparently contradicting hematological dogmas, that hematologists were hesitating in trusting it. The disease has been called hereditary hypofibrinogenemia with hepatic storage (HHHS).

## 3. Hereditary Hypofibrinogenemia with Hepatic Storage (HHHS)

**Figure 3:**
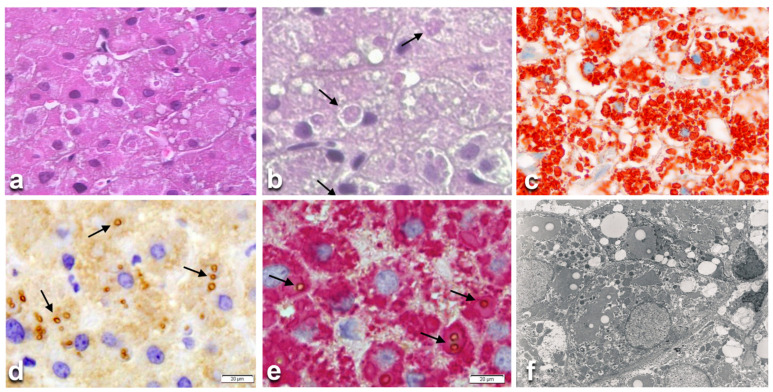
Histological liver section from a hereditary hypofibrinogenemia with hepatic storage (HHHS) patient. Hepatocytes contain round or polygonal eosinophilic cytoplasmic inclusions. Inside the eosinophilic inclusions there are single or multiple lipid vacuoles (**a**), H&E × 100). Lipid vacuoles are better visualized (arrows) in sections stained with PASD, despite their negativity to the stain. (**b**), PASD × 100). The eosinophilic PASD negative inclusions react positively (red color) to an anti-fibrinogen antibody (**c**), ×100). The lipid droplets inside the fibrinogen inclusions (arrows) are immunoreactive (brown color) to an anti-APO-B-lipoprotein antibody (**d**), ×100). Double immunostaining on the same section shows the co-localization of the two proteins (fibrinogen in red, APO-B- in brown marked by arrows (**e**), ×100). The EM picture shows lipid droplets within fingerprint-like fibrinogen aggregates. (**f**), EM × 10,000).

Light microscopy has been crucial in recognizing this disease, thereby providing a further solid bridge between pathology and molecular pathology. HHHS manifests in the liver and causes cirrhosis in early childhood. In histology, in H&E-stained preparation it is characterized by hepatocytic eosinophilic inclusions resembling AAT globules [2]. In contrast to AAT inclusion bodies, fibrinogen inclusions are negative or very faintly positive on PASD staining (Figure 3b) due to the very low carbohydrate content as compared to AAT. Fibrinogen inclusions are reacting exclusively and selectively with anti- fibrinogen antibodies (Figure 3c) [2,6,50].

Under the EM, fibrinogen inclusions appear mostly in the form of curved tubular structures arranged in a fingerprint-like fashion or elongated tubules with a fiber-glass configuration [2,50]. These features were first observed in two German families [51,52]. Full investigations were performed for the first time in an Italian patient undergoing cholecystectomy, duodenal polypectomy and a wedge liver biopsy because of an unexpected liver cirrhosis. On histological examination, the vast majority of hepatocytes contained round or polygonal eosinophilic (Figure 3a) PASD negative (Figure 3b) inclusions, positive on fibrinogen immunostaining (Figure 3c). On EM, the inclusions showed a fingerprint or a fiber glass like appearance. These inclusions were called fibrinogen type I inclusions [6] and have become the hallmark of HHHS. Other forms of fibrinogen inclusions in liver cells appearing as type II or III have been described as fibrinogen storage disease (FSD), but they are not associated with genetic alterations or low plasma level. Moreover, they are reversible in serial biopsies, thereby not fulfilling the criteria for ERSD [6]. The original Italian patient had very low fibrinogen plasma level but, surprisingly, no bleeding or coagulation problems or wound healing retardation. As several family members showed hypofibrinogenemia, the molecule was called fibrinogen brescia. The condition was named HHHS and the disease proposed as a new ERSD [2,6], in analogy with AATD [1].

Based upon the experience with AATD, a biochemical study on fibrinogen brescia was carried out at Max Planck Institute in Munchen in cooperation with Dr. Agnes Henschen. With the available technique, high performance liquid chromatography (HPLC), no amino acid replacement could be found in circulating fibrinogen brescia [53]. The disappointment was compensated eleven years later when the fibrinogen brescia DNA was sequenced in Christchurch in cooperation with Dr. Steve Brennan. A mutation in the gamma chain gene (FGG) (γ284Gly-Arg) was found and suspected to be responsible for the misfolding of the protein and for both hepatic retention and consequent hypofibrinogenemia. The examination of the purified brescia fibrinogen by electrospray ionization mass spectrometry explained the negative result obtained by HPLC as no abnormal γ chains were present in plasma [50]. Further studies on fibrinogen brescia with monoclonal antibodies against A α, B β, γ, E and D fragments could demonstrate that γ chain molecules were a major component of the aggregated fibrinogen within the RER. [54].

**Figure 4:**
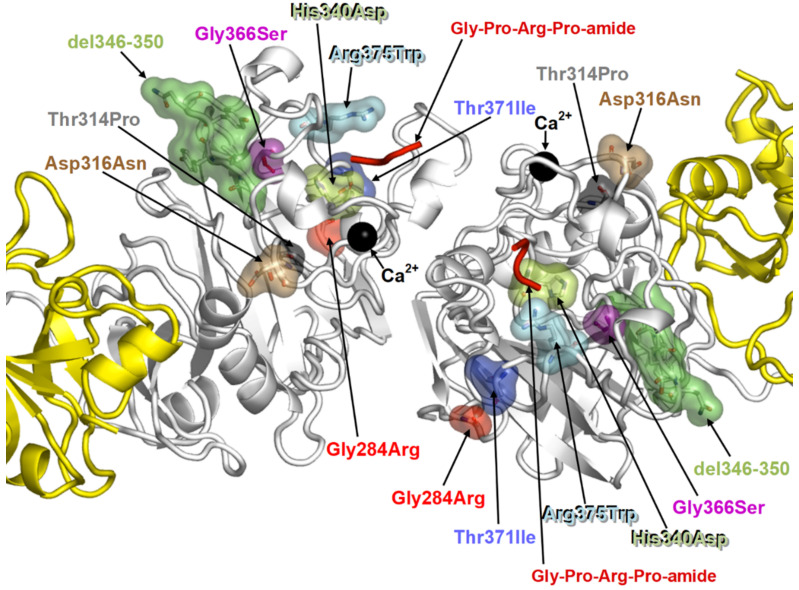
The 3D modelling shows the locations of the eight fibrinogen gamma chain mutations in the globular D-domain of the monomers that hamper the dimerization.

**Figure 5:**
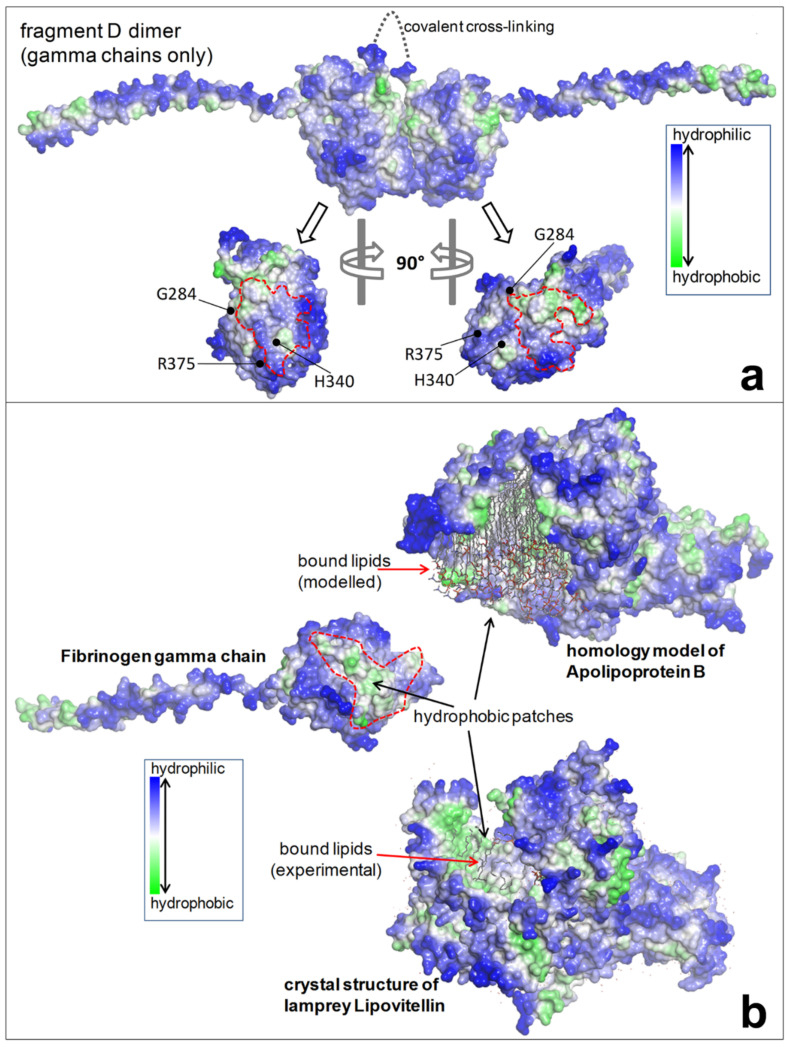
(**a**) The upper part shows the dimer fragment D (gamma chains only), covalently linked. The lower part shows the interaction sites (D-domain) of each monomer where three mutations (brescia, aguadilla, and ankara) are located. The mutations prevent interaction and hamper the D:D formation. (**b**) The failure of a correct polymerization leaves exposed hydrophobic patches in each monomeric mutant gamma chains which give rise to undue interaction with lipids and with hydrophobic regions of APO-B-lipoproteins and other lipids.

Following the discovery of fibrinogen brescia, and again starting from the observation in H&E-stained slides of eosinophilic PASD negative cytoplasmic inclusions in liver cells, six further mutations have been identified on the γ chain: aguadilla (Arg375Trp) [55], Al du Pont (Thr314Pro) [56], angers (G346_Q350del) [57], beograd (Gly366Ser) and pisa (Asp316Asn) [58], ankara (His340Asp) [59], and trabzon (Thr371Ile) [60]. The map of the fibrinogen gamma chain mutations on the structure of fragment double-D has been shown on 3D modelling (Figure 4) [60]. The eight mutations occur in the globular domain of the γ chain that is crucial for D dimers formation thus inducing important changes which hamper the gamma monomers interaction (Figure 5a) and cause its aggregation within the ER [7,59]. The calculations of the folding free energy changes show how the variants can affect the conformation and function, and has suggested the mechanism for the intracellular aggregation of all γ-module mutations causing HHHS [60].

Like in AATD, the aggregation of mutant fibrinogens occurs immediately after the discharge of the protein within the RER. However, in contrast with AATD in which the cell is capable of exporting some 15% of the mutant molecule, the eight mutant fibrinogen gamma chains, and the single case of a mutant alpha chain [61] cannot translocate to the SER and Golgi apparatus thus remaining entirely within the RER and are never exported.

The defective translocation from the RER to the other sections of the secretory pathway is documented by the absence of aggregated mutant proteins in the SER or Golgi apparatus, either in AATD or HHHS [6,22,50].

However, a small fraction of AAT mutants can escape the storage phenomenon, reach the circulation, and are detected on serum Isoelectric Focusing (IF). The mechanism of the escape is unknown. Starzl observed a reduction of the amounts of AAT storage in Pi Z cirrhotic patients after porto-caval shunt [62], thereby suggesting that a proportion of Z AAT could be progressively released into the circulation, albeit at a lower sped than normal protein [31]. This phenomenon is potentially relevant in view of the recent demonstration that ZZ AAT homopolymers can be secreted from cells through the canonical secretory pathway [63], or that heteropolymers (M or S and Z AAT) can be found in ER and in circulation [64].

Interestingly enough, recently, the formation of allopolymers of mutant fibrinogens and Apo-Beta lipoproteins has been described in patients with both hypofibrinogenemia and hypo-APO-beta-liproteinemia [7].

Mutant fibrinogens and Apo-beta-lipoproteins could be demonstrated in the same inclusions in both H&E (Figure 3a) and PASD (Figure 3b) stained preparations, in immunostained sections (Figure 3c,d) and in a double immunostained single section (Figure 3e) and by EM (Figure 3f). The molecular analysis has confirmed the mutations in the fibrinogen molecules whilst the sequencing of Apo-Beta encoding genes (APO-B and microsomal triglyceride transporter protein—MTTP) did not reveal any abnormality, thereby definitely indicating that the retention of APO-Beta was not genetically determined.

The analysis of the protein structures by 3D modelling in those cases has unraveled the pathomorphogenesis of the unexpected phenomenon. Fibrinogen gamma chain mutations indeed have been shown to provoke conformational changes in the region of the globular domain of the gamma chain (Figure 4), involved in the “end-to-end” interaction, thereby impairing the D-dimer formation (Figure 5a). Therefore, each monomeric fibrinogen gamma chain is left with an abnormal exposure of hydrophobic patches that become available for interactions with APOB and lipids (Figure 5b), causing their intracellular retention and impairment of export as a secondary unavoidable phenomenon [7].

Thus, the co-localization of two proteins simultaneously retained within the RER does not contradict the absolute requirement for ERSD (exclusive and selective retention of a mutant protein), as APO-beta-lipoprotein retention is not genetically determined but is an acquired phenomenon secondary to a physical-chemical affinity binding.

By analogy, the same holds true for AATD in which heteropolymers (M or S and Z) are entrapped within the RER, as recently demonstrated by confocal microscopy [64].

## 4. Conclusions and Perspectives

This review has confirmed the validity of the original definition of ERSD as an accumulation of abnormally conformed proteins due to mutations. The storage is exclusive and selective for the mutant protein and occurs at the site of the early synthesis, the RER. The condition is genetically determined, and hence congenital, hereditary, and permanent.

The mechanism of intracellular retention of mutant secretory proteins represents a basic phenomenon in biology and pathology and can potentially affect all secretory proteins.

Despite the low plasma level, these diseases are often missed in the clinic. The intracellular protein retention results in cytoplasmic inclusions that can be visualized by a light microscope on H&E-stained slides. The characterization of the inclusions requires stepwise investigations according to an ad hoc protocol.

The intracellular storage of mutant proteins causes cell constipation/damage/death, inflammation, and cirrhosis. The discovery of ERSD has led to the identification of the cause of a group of cirrhosis otherwise remaining cryptogenic. The unavoidable intracellular storage of mutant proteins represents the elementary lesion in ERSD, and consequently the true phenotype that strongly correlates with the genotype in both AATD and HHHS [60]. This statement helps in explaining why clinicians can miss individuals with AATD because they may not display serum deficiency or biochemical signs of liver disease, and HHHS individuals because they do not present overt signs of coagulopathy [60].

The diagnostic importance of H&E stain in general has been widely acknowledged in an Editorial (“thank you, H&E”) [65] by the great pathologist Juan Rosai who has been training generations of histopathologists worldwide over the last 60 years.

Hans Popper, the father of modern hepatology, often claimed that the best research tool is “H&E-stained slides connected to the brain” [66].

As fellows and great friends of the two above masters, we can testify to the fact that H&E that has fulfilled the requirements of modern medicine: (a) breaking the walls between the four watertight compartments of the traditional medicine (education, research, care, and laboratory services); (b) favoring the transition from the physician to the MD–researcher, and finally to PhD–scientist; (c) approaching difficult, rare, unknown cases with the habits and techniques previously prerogative of research alone, in this way turning a clinical case into a research project [67].

This background has made it possible for H&E stain to realize the miracle of forging the connection between the diagnostic histopathology of the past and the molecular pathology in the breve new world of the future.

It is astonishing that, running through various histological steps backwards and forwards from the most powerful techniques, i.e., IHC, in situ hybridization, EM, molecular histopathology, genetics, and 3D modelling, H&E, an old-fashion technique, properly observed with sensitive eyes, has begun again to tell us a story [67]. It has taken more than 50 years of histopathological research work to detect new mechanisms of disease and to explain the complete alpha and omega of the related conditions to the medical world.

In perspective, the body of cumulated knowledge should hopefully open the road to future strategies aimed to look for novel small molecules [68] capable of binding cleaved AAT or fibrinogen at the polymerized interface, to co-localize with AAT or fibrinogen gamma chain mutants, to inhibit intracellular accumulation and/or to increase their degradation.

## Figures and Tables

**Table 1:** Endoplasmic reticulum storage diseases (ERSD) and conformational diseases (CD).

A. Hepatic ERSD
Alpha-1-antitrypsin deficiency (AATD)
Heredirary Hypofibrinogenemia with hepatic storage (HHHS)
Alpha-1-antichymotrypsin deficiency (ACTD)
B. Extrahepatic CD
Synucleopathies:
Parkinson’s disease
Beta-amyloidosis
Dementia with Lewy bodies
Multiple system atrophy
Prion encephalopathy
C. Miscellanea
Alpha-2-antiplasmin inhibitor deficiency
C3 and C4 complement hepatic storage
Lebanese low density lipoprotein receptor defect
D. Others
Plasmacell Ig storage (Russell bodies)

## Data Availability

Not applicable.
